# The Impact of Surgical Wait Time and Hospital Stay on the Incidence of Burn Wound Infection and Related Complications at a Single Tertiary Hospital Centre: A 10-Year Experience

**DOI:** 10.1177/22925503241249756

**Published:** 2024-04-28

**Authors:** John Milkovich, Isabella F. Churchill, Lucas Gallo, Patrick Kim, Matteo Gallo, Achilles Thoma, Sophocles H. Voineskos, Cheryl Main, Christopher J. Coroneos

**Affiliations:** 1 Temerty Faculty of Medicine, University of Toronto, Toronto, ON, Canada; 2 Faculty of Medicine, University of Ottawa, Ottawa, ON, Canada; 3 Division of Plastic Surgery, 3710 McMaster University, Hamilton, ON, Canada; 4 Division of Plastic Surgery, University of Toronto, Toronto, ON, Canada; 5 Department of Pathology and Molecular Medicine, 3710 McMaster University, Hamilton, ON, Canada; 6 Hamilton Regional Laboratory Medicine Program, Hamilton Health Sciences, Hamilton, ON, Canada; 7 Department of Health Research Methods, Evidence and Impact, 3710 McMaster University, Hamilton, ON, Canada

**Keywords:** burns, burn unit, hospital stay, plastic surgery procedures, postoperative complication, time-to-treatment, wound infection, brûlures, unité des brûlés, séjour hospitalier, interventions en chirurgie plastique, complication postopératoire, temps d’attente avant le traitement, infection des plaies

## Abstract

**Introduction:** Admitted patients with burn injuries require prompt treatment and shorter hospital stays to avoid hospital-acquired infections and associated complications. This study aimed to determine the impact of time to the first surgery, and total length of hospital stay on the incidence of BWI and associated complications in adult patients with moderate burn injuries at a single tertiary hospital burn center. **Methods:** A retrospective chart review identified burn patients admitted to the Burn Unit between January 2011 and January 2021. The incidences of BWI and complications were documented. Univariate logistic regressions were performed, with significance set at *P* < .05. **Results:** A total of 171 patients were included in the study. The mean age was 50.8 years (range, 94-18 years), with 64 (37.4%) females. The mean wait time for surgery and total hospital stay was 2.6 days (SD = 2.5; 1-15 days) and 18.6 days (SD = 16.0; 1-125 days), respectively. Precisely, 47 patients (27.5%) were associated with positive burn wound cultures, with 23 patients (13.5%) experiencing clinical burn wound complications. Wait time to surgery did not significantly impact the incidence of BWI (OR = 0.97, *P* = .72) or related complications (OR = 0.97, *P* = .61). Total hospital stay was significantly associated with the incidence of BWI (OR = 1.05, *P* < .001) and related complications (OR = 1.02, *P* = .03). **Conclusions:** Delay to surgery may not be a significant risk factor of BWI and related complications in patients with burn injuries. Half of positive burn wound cultures are associated with objective clinical infection and subsequent complications. However, total hospital stay may be clinically important.

## Résumé

**Introduction:** Les patients hospitalisés à cause de brûlures doivent être traités rapidement et être hospitalisés peu longtemps pour éviter les infections nosocomiales et les complications connexes. La présente étude visait à déterminer l’effet du temps d’attente jusqu’à la première opération et du séjour hospitalier total sur l’incidence d’infection des plaies par brûlure (IPB) et des complications connexes chez des patients adultes ayant des plaies par brûlure modérées dans un seul centre hospitalier de soins tertiaires. **Méthodologie:** Les chercheurs ont procédé à une analyse rétrospective des dossiers hospitaliers et extrait les cas de patients brûlés admis à l’unité des brûlés entre janvier 2011 et janvier 2021. Ils ont consigné l’incidence d’IPB et de complications et procédé à des régressions logistiques univariées, dont la signification était établie à *P* < 0,05. **Résultats:** Au total, 171 patients, d’un âge moyen de 50,8 ans (plage de 18 à 94 ans) ont été inclus dans l’étude, y compris 64 femmes (37,4%). Le temps d’attente moyen avant l’opération et la durée totale du séjour hospitalier étaient de 2,6 jours (ÉT = 2,5; 1 à 15 jours) et de 18,6 jours (ÉT = 16,0; 1 à 125 jours), respectivement. Dans l’ensemble, 47 patients (27,5%) ont obtenu un résultat positif à la culture des plaies par brûlure, et 23 patients (13,5%) ont présenté des complications cliniques. Le temps d’attente jusqu’à l’opération n’a pas eu d’incidence importante sur l’IPB (RC = 0,97, *P* = 0,72) ni sur les complications connexes (RC = 0,97, *P* = 0,61). Le séjour hospitalier total était associé de manière significative à l’incidence d’IPB (RC = 1,05, *P* < 0,001) et de complications connexes (RC = 1,02, *P* = 0,03). **Conclusions:** Le temps d’attente avant l’opération n’est peut-être pas un facteur de risque important d’IPB et de complications connexes chez les patients brûlés. La moitié des résultats positifs aux cultures des plaies était associée à une infection clinique objective et à des complications subséquentes. Cependant, la durée totale du séjour hospitalier pourrait avoir une importance clinique.

## Introduction

Burn injuries are a considerable cause of morbidity and mortality, which heavily weigh on healthcare systems. According to data collected by the American Burn Association National Burn Repository (ABA-NBR) between 2009 and 2018, 221 519 acute burn admissions were recorded across 101 accredited North American hospitals.^1^ Overall, burn patients require hospitalization for 8.5 to 10 days, with 3% global mortality.^1^ In subsets of severe burns affecting 50% of total body surface area (TBSA), mortality rates can be as high as 40%.^1^ The economic burden of burn injuries on healthcare systems is significant.^1^ In 2010, the total injury cost associated with fire/burn incidents in Canada was $366 million.^2^ This sum can be subdivided into $177 million attributed to direct healthcare expenses and $188 million associated with indirect costs from factors including diminished productivity, disability, and premature death.^2^

The potential of a wound infection at the burn site introduces complexity in burn care.^1^ The mortality rate of burn patients with infection is twice that of those without infection due to burn-related sepsis,^3,4^ which is responsible for 42% to 65% of deaths from burn injury.^5–8^ Among patients with severe burns over 40% TBSA, sepsis from burn wound infection causes 75% of all deaths.^9^ Burn wound infection from nosocomial transmission is the most common; previously identified risk factors include delayed wound excision, TBSA, and burn severity.^4,10^ The advent of multidrug-resistant bacteria species such as *Pseudomonas* spp., *Acinetobacter baumannii*, *Stenotrophomonas maltophilia*, and *Staphylococcus aureus* exacerbates this health crisis.^11^ One Canadian study by Wanis et al found that the proportion of multidrug-resistant bacteria species isolated from burn infections increased from 6% at 1 week to 44% after 28 days of hospitalization.^12^

Various programmatic interventions, such as monitored handwashing, early removal of urinary catheters, and frequent changes of central lines, have been designed to reduce the incidence of nosocomial burn wound infections.^13^ However, there is a paucity of evidence regarding how wait time for surgery affects the risk of these infections. It is hypothesized that longer wait times increase patient exposure to pathogens in the hospital while in an untreated vulnerable state, thereby elevating their risk of contracting a burn wound infection. As such, the primary objective of this study is to determine the relationship between wait time to surgery, defined as the period between the time of patient consent to surgery, and culture-positive burn wound infections and associated complications among adult patients with moderate burn injuries. The secondary objective is to assess how the total length of hospital stay impacts the risk of culture-positive burn wound infections and associated complications. While the treatment of burn wound infections typically confers prolonged hospital stay, we hypothesize that this relationship is bidirectional such that increased hospitalization simultaneously increases patients’ exposure to infection.

## Methods

### Study Design and Sample

The investigators conducted a retrospective observational study of all patients admitted to the Hamilton Health Sciences Centre Burn Unit (BU) at the Hamilton General Hospital with a split provincial catchment population of about 7 million people. Patient information was gathered from electronic medical records in a single network in Hamilton, Ontario, Canada, from January 1, 2011 to January 1, 2021.

### Data Collection

Patients were identified by ICD-10 diagnosis codes formed with provincial burn reporting data. A retrospective chart review was performed of their medical records to collect data on all adult patients (ie, ≥ 18 years of age) who underwent surgery for burns involving < 20% of their TBSA between January 1, 2011, and January 1, 2021. Patients who provided consent on the same day as their surgery (ie, wait time to surgery < 1 day) or who had missing information were excluded from the study. Large TBSA burns were excluded given the priority of their care; these patients did not wait for intervention given their burn injuries were emergent. If patients received multiple surgeries, only the surgery associated with the primary burn admission was analyzed. We extracted relevant demographic and procedural information by reviewing patient records, which included preoperative consent forms, operative reports, and clinical charts. This information was then collated into a database.

### Patient Variables

Patient variables were recorded for all patients in the following categories: (1) demographic information (age, gender, smoking status, length of stay, and diabetes status); (2) details of the burn injury (including burn site, TBSA, third-degree full thickness, and etiology); and (3) information about treatment (such as time to first surgery, number of surgeries, type of operation performed, presence of culture-positive burn wound, and occurrence of complication).

The primary predictor variable was the time to the first surgery, which we measured as the number of days between the date that patient consent for surgery was obtained and the date of the first surgery. Importantly, the time of consent is a proxy for when surgery was clinically indicated and therefore the healthcare team obtained consent. In a practical sense, patients may encounter surgical delays stemming from bottlenecks within Canada's surgical priority queue, warranting patient agreement for surgery as an appropriate proxy for wait time from a system perspective. Secondarily, the length of hospital stay was calculated by determining the number of days between the date of admission and the date of discharge.

### Outcome Variables

The primary outcome variables of this study are the presence of a culture-positive burn wound infection (BWI) in the context of clinical suspicion of infection. To test for wound colonization, we obtained swab cultures were obtained and their species type and resistance were identified; these tests were not routine in our BU during the study period. BWI-associated complications were defined as cases with a culture-positive BWI, and where clinical wound infection was specifically observed and noted in the patient's chart. We were specific in excluding complications involving systemic issues while including cases of cellulitis, fever, bacteremia, graft failure, and contracture.

### Statistical Analysis

Descriptive statistics were used to summarize patient demographic and clinical characteristics. Continuous variables were presented as means and ranges, while discrete variables were presented as raw numbers and proportions. To evaluate the primary objective, patient data was divided into 4 categories: those with complications versus those without complications, and those with culture-positive BWIs versus those with culture-negative burn wounds.

To test the null hypothesis that the median time to the first surgery was the same for both groups, a nonparametric Mann-Whitney U-test was performed. This statistical test was chosen after testing for the normality and equal variances assumptions of the data using the Shapiro-Wilk and Levene's tests, respectively. Univariate logistic regressions were performed for both the primary and secondary objectives to estimate the probability of a binary event (ie, complication and culture-positive BWI). Time to the first surgery and total length of hospital stay were included as the continuous predictor variables. The beta coefficient estimates were exponentiated to obtain the odds ratios, and 95% confidence intervals and *P*-values were calculated. An alpha of 0.05 was considered significant. All statistical analyses were conducted in Jupyter Notebook (Version 6.4.5)^14^ with the Python programming language (Version 3.9.7).^15^

## Results

During the 10-year period, 707 burn patients were admitted to the BU; however, only 171 patients met the study's inclusion criteria. The mean age of the patients was 50.8 years (range, 94-18 years), with 64 (37.4%) females. The mean wait time for surgery was 2.6 days (SD = 2.5; range, 1-15 days), and the mean length of hospital stay was 18.6 days (SD = 16.0; range, 1-125 days).

The average TBSA of the burn injuries was 9.0% (range, 0-20). Among these, 105 (61.4%) were diagnosed as third-degree full-thickness burns, and 15 (8.8%) cases required mechanical ventilation due to inhalation injury. Detailed demographic, procedural information, and characteristics of the burn injuries in our study's cohort, including burn site and etiology, are shown in Table 1.

**Table 1.** Patient Demographics and Procedural Characteristics (n = 171).

Patient demographic/procedural and burn characteristic	Mean (range)/number (%)
Age (years)	50.8 (18-94)
Sex	Male: 107 (62.6%)
	Female: 64 (37.4%)
Smoking status	Never: 88 (51.5%)
	Former: 8 (4.7%)
	Current: 75 (43.9%)
Diabetes status	None: 148 (86.5%)
	Type 1: 4 (2.3%)
	Type 2: 19 (11.1%)
Type of surgery	Debridement only: 9
	Debridement and skin graft: 192
	Debridement and flap reconstruction: 2
Length of hospital admission (days)	18.6 (1-125)
Time to first surgery (days)	2.6 (1-15)
Length of surgery (minutes)	153 (47-425)
Postoperative length of stay (days)	13.2 (0-121)
Total number of surgeries	1.2 (1-4)
Burn site	Head and neck: 50 (29.2%)
	Upper limb: 113 (66.1%)
	Lower limb: 78 (45.6%)
	Trunk: 88 (51.5%)
Total burn surface area (%)	9.0 (1-20)
Burn etiology	Scald: 29 (17.0%)
	Flame: 100 (58.5%)
	Chemical: 4 (2.3%)
	Electrical: 3 (1.8%)
	Contact: 35 (20.5%)
Inhalation Injury w/mechanical ventilation	15 (8.8%)
TBSA third degree full thickness	105 (61.4%)

Abbreviation: TBSA, total body surface area.

Out of the 171 patients, 47 (27.5%) developed culture-positive BWIs, and 23 (13.5%) experienced complications associated with these BWIs (Table 2).

**Table 2.** Primary Outcomes of All Patients (n = 171).

Outcome	Mean (range)/number (%)
Culture-positive burn wound infection	47 (27.5%)
Complications related to burn wound infection	23 (13.5%)

### Time to the First Surgery

The relationship between the time to the first surgery and length of hospital stay versus culture-positive BWIs and complications are summarized in Table 3.

**Table 3.** Wait Time to First Surgery and Length of Hospital Stay Versus the Presence of Burn Wound Infections and Associated Complications.

	Status	No. (%)	Mean (SD) (days)	*P* value
Wait time to first surgery				
Infection	Culture-positive	47	2.5 (2.6)	.37
	Culture-negative	124	2.7 (2.4)	
Complication	Yes	23	2.5 (1.8)	.79
	No	148	2.7 (2.6)	
Length of hospital stay				
Infection	Culture-positive	47	27.2 (22.2)	<.001*
	Culture-negative	124	15.4 (11.5)	
Complication	Yes	23	26.1 (15.2)	<.001*
	No	148	17.5 (15.9)	

* Significant at *P* < .05.

There was no significant difference between patients who developed a culture-positive BWI compared to those who did not (MD = 0.2 days, *P* = .37), as well as patients who developed an infection-related complication compared to those who did not (MD = 0.2 days, *P* = .79).

**Figure 1.**
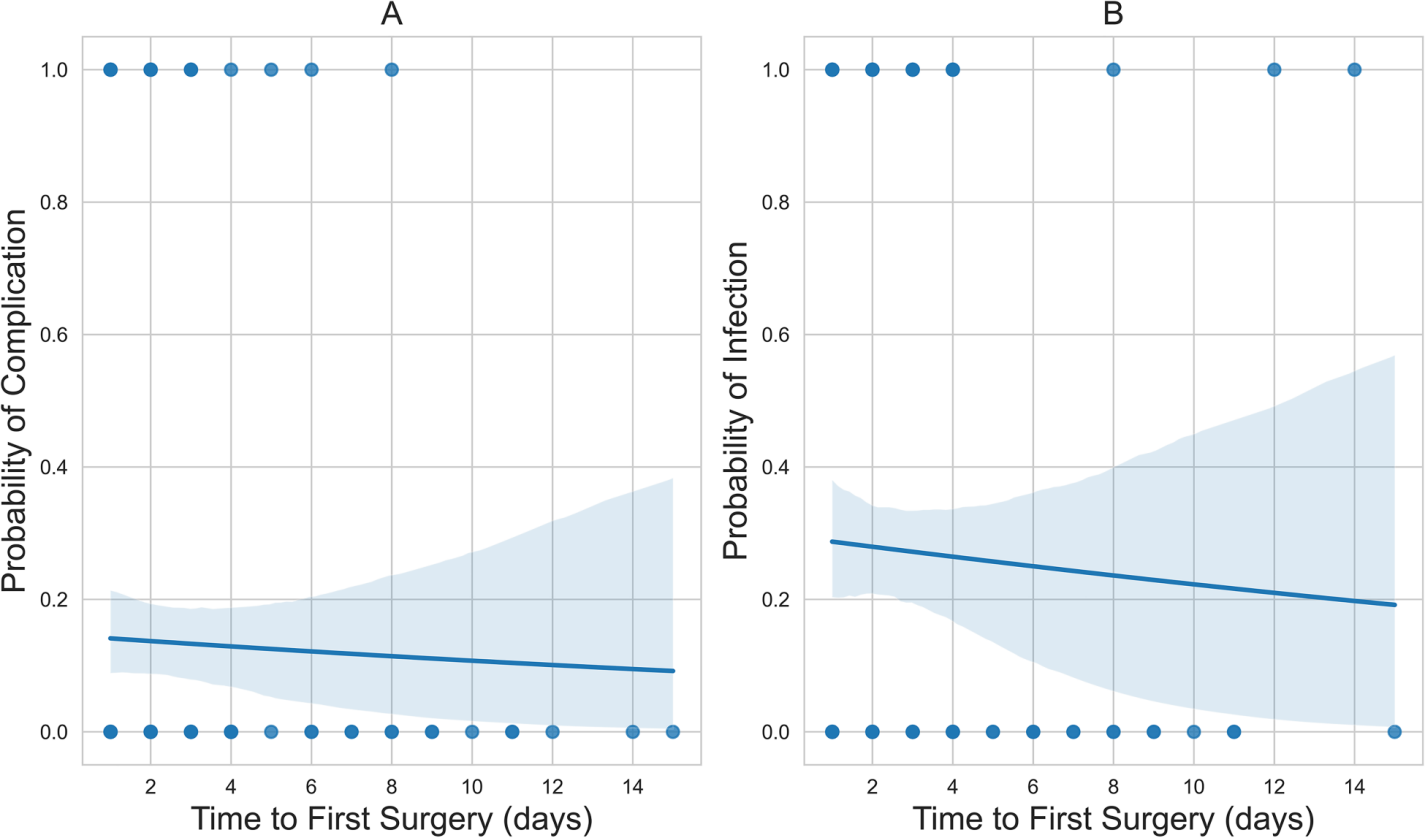
(A) The downward-sloping probability curve of time to first surgery versus related complication; and (B) the downward-sloping probability curve of time to first surgery versus culture-positive wound infection status.

There was no significant association between time to first surgery and BWIs (OR = 0.97, *P* = .72) and associated complications (OR = 0.96, *P* = .61) (Table 4). The probability curves in Figure 1 represent the logistic regression probability curves.

**Table 4.** Logistic Regression Odds Ratios of Time to First Surgery and Total Hospital Stay Versus Adverse Patient Outcomes.

	OR (95% CI)	*P* value
Wait time to first surgery		
Culture-positive burn wound infection	0.97 (0.80-1.17)	.72
Burn wound infection-related complication	0.96 (0.83-1.11)	.61
Length of hospital stay		
Culture-positive burn wound infection	1.05 (1.02-1.08)	<.001*
Burn wound infection-related complication	1.02 (1.00-1.05)	.03*

* Significant at *P* < .05.

### Total Length of Hospital Stay

Those with a culture-positive BWI stayed in the hospital for a mean of 11.8 more days than those who did not develop a culture-positive BWI. Similarly, those who developed a complication from their BWI were hospitalized for a mean of 8.6 more days than those who did not.

**Figure 2.**
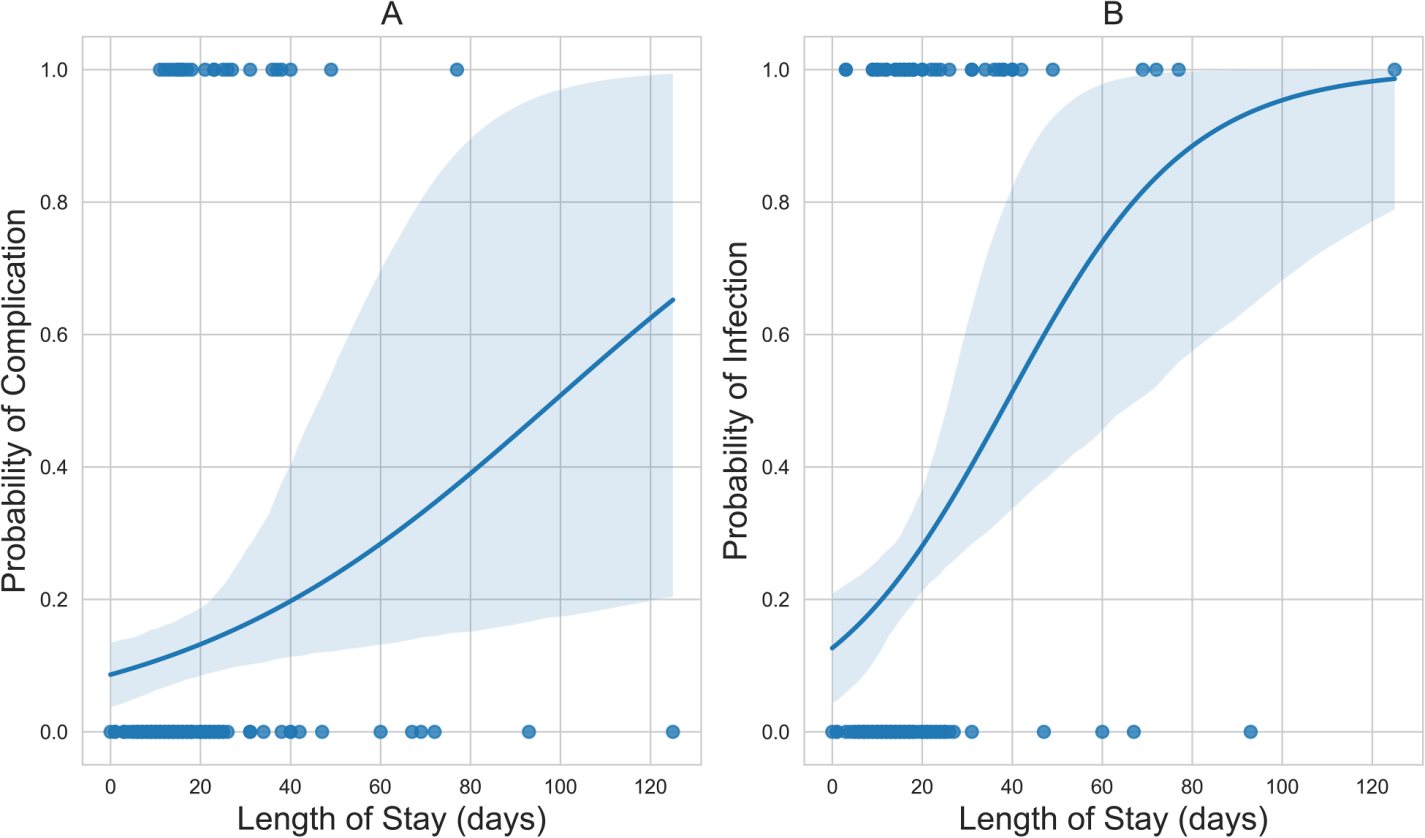
(A) The positive sigmoid probability curve of hospital stay versus complication; and (B) the positive sigmoid probability curve of time to first surgery versus culture-positive wound infection status.

Total hospital stay was associated with an increased incidence of BWIs (OR = 1.05, *P* < .001) and associated complications (OR = 1.02, *P* = .03) (Table 4). The corresponding probability curves for the logistic regression models are presented in Figure 2.

## Discussion

In this article, we highlight the impact of wait time for surgery and length of hospital stay on the incidence of culture-positive BWIs and related complications at a single tertiary burn center. Further, we establish a correlation between culture-positive BWIs and clinical infection. Over 10 years, 27.5% of patients had a culture-positive BWI, of which 48.9% of them developed associated complications. Contrary to our initial hypothesis, wait time for surgery had no statistically significant impact on the incidence of culture-positive BWI (OR = 0.96, *P* = .72) and related complications (OR = 0.96, *P* = .61). However, our analysis showed that patients with culture-positive BWIs were hospitalized for an average of 11.8 more days than patients without infection. As such, there was a positive relationship between the length of hospital stay and the incidence of culture-positive BWI (OR = 1.05, *P* < .001) and related complications (OR = 1.02, *P* = .03).

Our findings build upon the evidence from previous studies with similar designs. A 10-year study at a single university burn center in Turkey found delayed burn wound excision to be a significant factor in the development of infection (OR = 1.14, *P* < .001).^4^ While the results of our study did not support their findings, Alp et al defined delayed burn wound excision as “First-day excision” and included patients with TBSA ranging from 0% to 99%. In contrast, the scope of our study focused only on patients with moderate burns (TBSA < 20%) treated beyond the same day of patient consent for surgery. Therefore, our findings do not necessarily contradict those of Alp et al but instead suggest that the effect of a longer delayed BWI is less substantial for burn injuries with TBSA < 20%. Another study based at a single burn unit in China examined the impact of hospital stay duration on the occurrence of infection.^10^ In agreement with our results, patients without BWIs had an average hospital stay of 13 days, while patients with infections stayed for an average of 27 days (in contrast, our study found average hospital stays of 15.4 and 27.2 days, respectively). In addition, they demonstrated a positive correlation between infection and extended length of hospital stay among burn patients.

It is important to note, however, that the positive association between length of stay and culture-positive BWI and complication rates in our study does not prove causation. It is challenging to disentangle the effect of the length of hospital stay on BWI, or vice versa. This relationship is complex and bidirectional such that patients may experience an extended hospital stay because of the additional treatment required to address preexisting BWIs or other factors that were not acquired in the hospital setting. More specifically, patient factors such as age, third-degree TBSA%, inhalation injuries, number of procedures performed, sepsis, graft loss, days ventilated, and in-hospital complications such as bacteremia, pneumonia, sepsis, graft loss, and respiratory failure were associated with a longer-than-expected length of stay in a 10-year retrospective cohort study conducted at a single adult burn-center in Toronto, Canada.^16^ We are therefore not in a position to draw conclusions about nosocomial infections because we did not routinely collect admission cultures, which would clarify how many BWIs were acquired in the hospital versus those present in the external environment at the time of admission. Further research is needed to establish the dominant forces in this complex and multifactorial relationship to inform the optimization of burn care.

The modernization of burn centers has led to the incorporation of several programmatic interventions aimed at controlling the transmission of infections, which have been further reinforced as part of hospital protocol in response to the COVID-19 pandemic. A study by Hultman et al^13^ highlighted that the incidence of BWIs (measured as infection rate per 1000 patient days) decreased from 11.7 to 6.5 over 10 years as infection control measures were incrementally introduced across US burn centers. These improvements included semi-quarantining patients with multiple drug-resistant hospital-associated infections, utilizing full barrier precautions for bedside procedures, changing central lines every 3 days, early removal of urinary catheters, monitoring handwashing, and performing broncho-alveolar lavage on patients with ventilator-assisted pneumonia. In addition, the Enhanced Recovery after Surgery (ERAS) protocol is an evidence-based multimodal strategy that has been recently popularized and is being continuously implemented across surgical specialties as a means to lower recovery time and reduce postoperative complications. ERAS protocols achieve these objectives through the optimization of perioperative management, from admission to discharge. Such a protocol has been proposed by Sljivic et al^17^ for burn care. Preoperatively, they recommend including implementing acetaminophen and pregabalin/gabapentin on-call to the operating room, whereas postoperatively, they advise that the healthcare team periodically check lactate levels, maintain an operating room temperature at 85 °F, and continue ketamine infusion for burns >30% TBSA. It is certainly worthwhile to continue to investigate the effects of ERAS on burn care given its complexity, predisposition to other complications, and overall burden on the healthcare system.

On that note, burn injuries pose a massive economic burden relative to all plastic surgery-related diseases in Canada. In 2019, fire, heat, and hot substance injuries ranked as the third most expensive contributor to age-standardized cost, amounting to 1.2 CAD billion behind squamous cell carcinoma (1.3 CAD billion) and breast cancer (5.1 CAD billion).^18^ This hefty cost is justified by its high mortality and morbidity rates of burns, thereby yielding the greatest return on investment and funding allocation. However, the authors of this study suggest that further economic analysis should be performed on traumatic burn injuries to advocate for more operating rooms and minor procedure time to make surgical treatment more accessible and timelier. While we support this endeavor, we would like to point out that our findings are encouraging as they suggest that the hospital protocols currently in place at our BU have effectively allowed patients to receive timely surgical treatment of their burns without increasing their risk of in-hospital infection or complication, thereby improving the quality of care and avoiding the additional costs of treating associated complications stemming from prolonged delays in surgery.

There are several limitations to this study. Due to its retrospective nature, these findings cannot prove causation. This is especially relevant for the findings of the secondary objective that suggest a positive relationship between the total length of hospital stay and the occurrence of BWIs and related complications. In addition, we used the time between patient consent and first surgery as a proxy to wait time to surgery. This surrogate metric for time to first surgery is a limitation given that we opted to exclude all patients who provided consent on the same day of surgery due to the inability to track the number of hours between patient consent and surgery. Alternatively, by including all patients with 0-day wait times, it is likely that this would inappropriately skew the data and conceal the relationships of interest in this study. The trade-off of this exclusion criterion is that we omitted patients from our cohort, which limited the sample size of this study. Furthermore, the availability of the data was also limited by the retrospective design, which prevented additional analysis on other pertinent variables, such as the extent of the full versus partial thickness burns of the total TBSA for third-degree burns. Lastly, we equated culture-positive burn wounds to BWIs, which is a diagnostic overextension given initial burn wound colonization and not yet clinical infections. To clarify this, we stated that half of positive burn wound cultures are associated with objective clinical infection and subsequent complications.

## Conclusion

Overall, this 10-year experience provided important insights into the impacts of wait time to surgery and total hospital stay on the incidence of BWIs and related complications among patients with moderate burn injuries. Half of positive burn wound cultures are associated with objective clinical infection and subsequent complications. Research on this topic is limited, and thus this study fills a critical knowledge gap.
